# Supplementary material for: Bond strength of thermoformed and 3D-printed aligners with universal primer versus one-step aligner adhesive with and without sandblasting: An in vitro study
Source: PLoS One. 2026 Jan 23;21(1):e0341664. doi: 10.1371/journal.pone.0341664 (PMC12829832; doi:10.1371/journal.pone.0341664)
Supplement: Attachment — Submitted filename: review.docx [file pone.0341664.s002.docx]

The paper titled ‘ Bond strength of thermoformed and 3D-printed aligners with universal primer versus one-step aligner adhesive with and without sandblasting: an in vitro study” is of good scientific quality and well structured. However, some critical points need to be clarified and further discussion is required before acceptance. As follows:

1- The statement that “Published evidence is virtually confined to one study” require justification with more references. Were there truly no other related studies? Add more references to support your claim.
2- What is the clinical siginificance of direct bonding to alignes VS enamel?
3- In the methodology, there are some areas need clarifications

- Please provide a schematic diagram or flow chart of the sample preparation and grouping for clarification.
- Why only alumina sandblasting used? Explain the reason for selecting this type of surface treatment.
- Why 500 thermocycles were selected?

4- Results are comprehensive, but:

- table 4 is difficult to follow
- Bond strengths is very high. Any comments?
- “Failure mode was material-dependent and not completely parallel with bond strength”. Please explain.
- Figures (2-5) need further explaniations.

5- In the discussion, clinical translation could be expanded.
6- The language is good and overall clear but some paragraphs in the discussion need to be shorten.
7- Ensure consistent use of terminology: eg., Bond aligner or aligned adhesive.

Recommendation: Minor to moderate revision. The manuscript is strong and novel but require clarification in methodology, results and discussion.
